# foodMASST a mass spectrometry search tool for foods and beverages

**DOI:** 10.1038/s41538-022-00137-3

**Published:** 2022-04-20

**Authors:** Kiana A. West, Robin Schmid, Julia M. Gauglitz, Mingxun Wang, Pieter C. Dorrestein

**Affiliations:** 1 Collaborative Mass Spectrometry Innovation Center; University of California San Diego, La Jolla, CA 92093 USA; 2 Skaggs School of Pharmacy and Pharmaceutical Sciences; University of California San Diego, La Jolla, CA 92093 USA; 3 Center for Microbiome Innovation, Joan and Irwin Jacobs School of Engineering; University of California San Diego, La Jolla, CA 92093 USA; 4 Department of Pediatrics, School of Medicine, University of California San Diego, La Jolla, CA 92093 USA

**Keywords:** Health sciences, Mass spectrometry

## Abstract

There is a growing interest in unraveling the chemical complexity of our diets. To help the scientific community gain insight into the molecules present in foods and beverages that we ingest, we created foodMASST, a search tool for MS/MS spectra (of both known and unknown molecules) against a growing metabolomics food and beverage reference database. We envision foodMASST will become valuable for nutrition research and to assess the potential uniqueness of dietary biomarkers to represent specific foods or food classes.

## Main

Society is becoming more conscious of the foods we consume and more interested in understanding the compositions of specific dietary components. This sentiment is driven by the increasing awareness that diet influences our general health, including our microbiome, immune homeostasis, and even cognitive function. At the same time, our capacity to leverage untargeted metabolomics data, where many unidentified mass spectrometry signals are observed, has dramatically increased with the development of computational ecosystems such as GNPS/MassIVE^1^. There are now ~150 cataloged and tracked nutritional components, but a large proportion of the chemicals present in foods are unreported making it hard to assess if they are unique to the foods being studied^2^; it remains impossible to determine the uniqueness of these molecules across different foods and food groups.

**Fig. 1:**
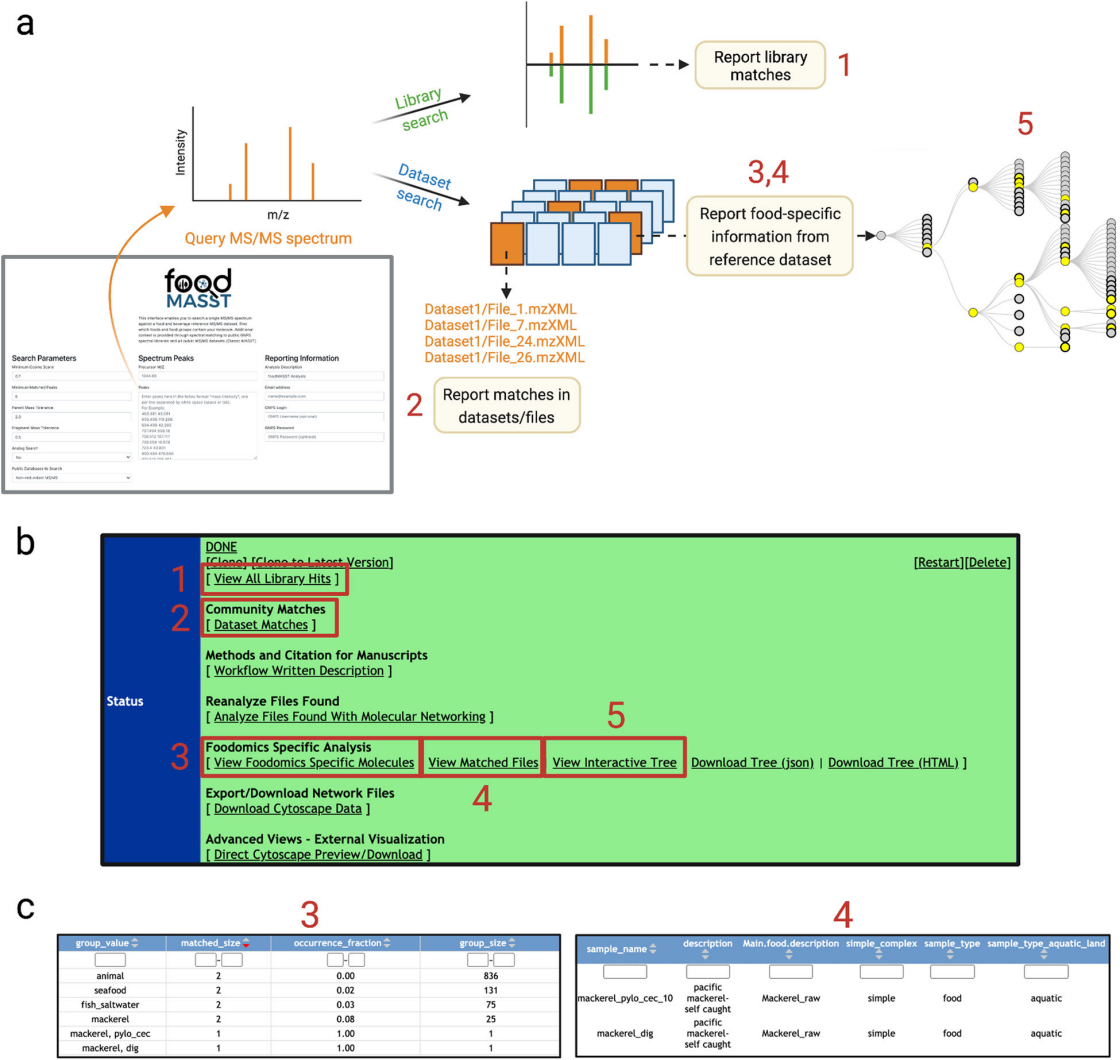
foodMASST workflow and reports. **a** A foodMASST query will search both library MS/MS spectra and metabolomics datasets that have been deposited to GNPS/MassIVE. **b** The results page lists various statistics **c** and further analysis options. Library spectra that match the query are reported (1) as potential annotations if the molecule is unknown. Public datasets, and files within datasets, containing spectral matches to the query are also reported (2) to provide context about where the molecule is observed. Additional reporting based on matches within the food reference dataset can be viewed and downloaded under “Foodomics Specific Analysis”. The percentage of samples containing a spectral match for each category of the food ontology are tabulated (3). Metadata for each food item with matches is reported (4). A visual summary of the food ontology and matches in each category can be viewed in the browser (5) or downloaded.

The Mass Spectrometry Search Tool (MASST)^3^ combined with a reference database of food metabolite data can be a powerful resource to understand the molecular landscape of foods. MASST is a mass spectrometry search engine that identifies all data files in the GNPS/MassIVE untargeted metabolomics repository that contain a spectral match to a query MS/MS spectrum. We created a domain-specific MASST, called foodMASST (https://masst.ucsd.edu/foodmasst), to enable reporting of the search results in the context of foods and beverages (Fig. 1a). As of Feb 2021, ~3500 untargeted metabolomics files from different foods/beverages collected as part of the Global FoodOmics Project^4^ (GFOP) have been deposited in MassIVE, a public mass spectrometry repository. Each item is classified according to a customized food ontology and annotated with additional metadata (e.g., raw or cooked, cooking method, place of origin). FoodMASST utilizes this reference dataset to determine the food/beverage items containing a query spectrum and to contextualize the molecule’s presence across foods. To increase usability by others in the community, we created a web interface to launch searches of known and unknown molecules with user-defined parameters and report the food information associated with the fragmentation data (MS/MS) matches. Confidence in spectral matches can be controlled by tuning the search parameters. Although powerful and useful nutrition-related search tools such as FooDB (www.foodb.ca), KNApSAcK, and others^5–8^ exist, foodMASST is the only tool to enable spectral matching of both known and unknown molecules in simple (single ingredient) and complex foods and beverages.

To start a foodMASST job, the parent mass and the MS/MS spectrum details are entered into the web interface. Although the reference data that is currently searched was collected with ramped collision energies on a Q-TOF, and searching with data collected in a similar manner will give the best results, to enable searching with data that is collected differently (e.g., different collision energies, more or less background noise, and from different instruments), the data is filtered by keeping the top 6 ions in +/−50 Da window (with a 100 Dalton effective sliding window) and subjected to a square root before searching. With the cloud-based platform GNPS, all results are sharable with provenance and tied to user accounts. Once a job is completed, the results can be navigated through the landing page which displays several links to reports (Fig. 1b). To provide additional context automatic spectral library search is performed against more than 30 public spectral libraries in the GNPS/MassIVE ecosystem including GNPS contributed libraries, Human Metabolome Database^9^, all three Massbanks^10–12^, and many others (for a list see https://gnps.ucsd.edu/ProteoSAFe/libraries.jsp) to determine if molecules were known. “Dataset matches” navigates to all datasets (and files within those datasets) containing matches to the query spectrum. Reports specific to foodMASST can be found under “Foodomics Specific Analysis”. For each category in the food ontology, the proportion of matches are reported in “View Foodomics Specific Molecules” and visualized in “View Interactive Tree”. Metadata associated with the matching foods is reported in “View Matched Files”. For example, when the MS/MS library spectrum for domoic acid, a potent neurotoxin from dinoflagellate blooms in the ocean^13^, was searched (parent mass tolerance: 0.1 Da, ion tolerance: 0.1 Da, minimum matched peaks: 6, cosine score threshold 0.65; see data availability for job link), the only two matches to the FoodOmics reference data obtained were associated with seafood—freshly caught mackerel (Fig. 1c). It is important to consider that isomers often share similarity in their MS/MS spectra and the user should interpret the results with this in mind. If there are multiple spectral matches possible within the scoring criteria, all spectral annotations are provided. For example, an MS/MS spectrum may match to spectra with the annotation of L-leucine, D-leucine, L-isoleucine, D-isoleucine as MS/MS-based mass spectrometry usually does not provide regiochemical and stereochemical information.

**Fig. 2:**
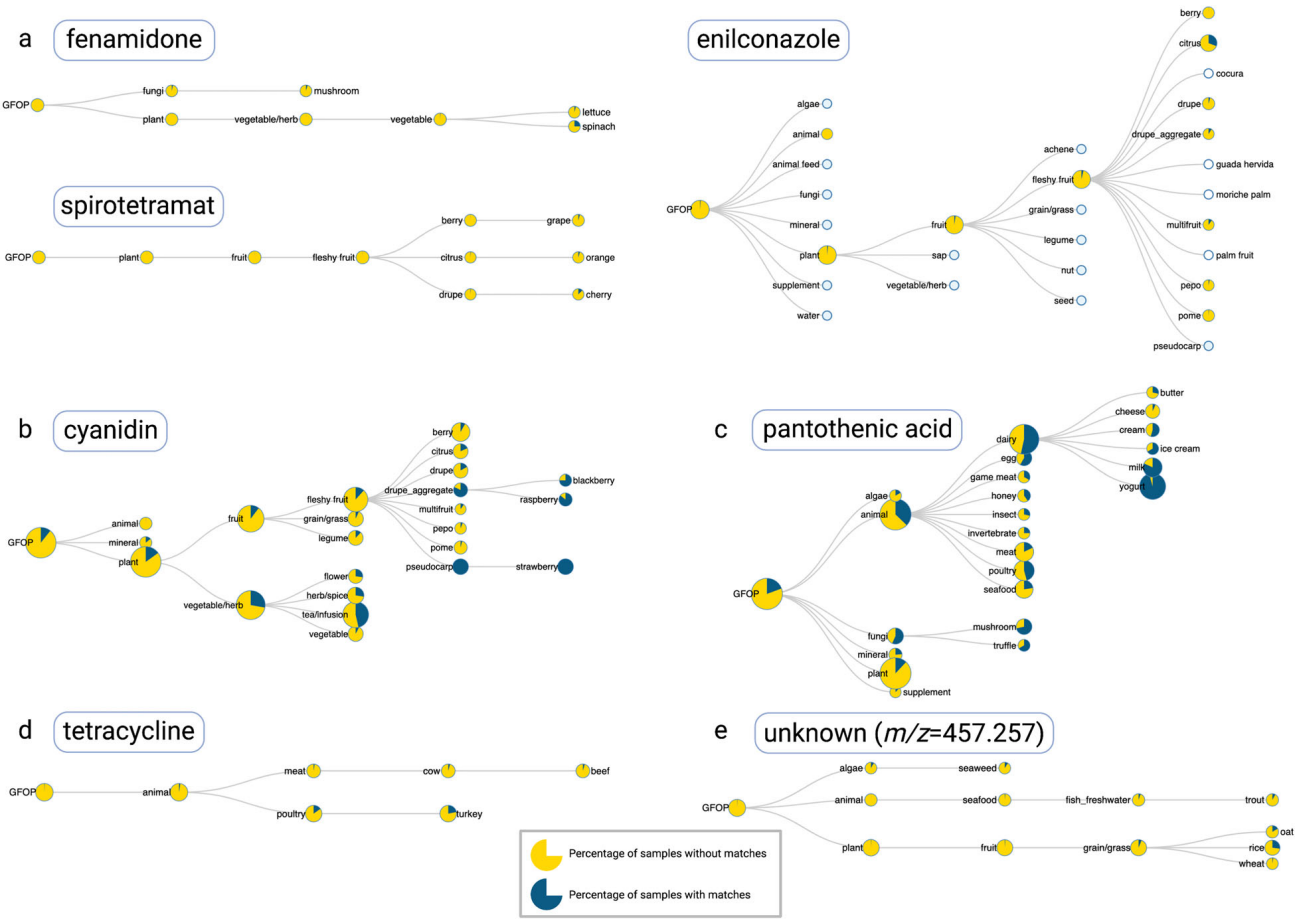
Examples of foodMASST results. **a** Three biocides were queried against the Global FoodOmics reference dataset to determine their presence among sampled foods. **b** Cyanidin, a plant pigment responsible for reddish-purple color, was observed in the expected categories such as teas, blackberries, raspberries, and strawberries. **c** Vitamin B_5_ was observed across many food categories but was most prevalent in animal and fungi samples. **d** Tetracycline, an antibiotic commonly administered to livestock, was detected only in beef and turkey samples. **e** An unknown molecule detected in biospecimens from Alzheimer’s patients may be related to the consumption of oats or rice. Nodes are scaled according to the total number of samples classified for that ontology term or any of its descendants. Pies represent the percentages of samples with (blue) and without (yellow) matches to the query spectrum.

We performed additional MS/MS searches for 6 library standards and one unknown compound. These included: biocides fenamidone, spirotetramat, and enilconazole; the plant pigment cyanidin; Vitamin B_5_; the antibiotic tetracycline; and an unknown molecule with a precursor *m/z* of 457.257. Fenamidone is a fungicide with low use in the US^14^ and accordingly was detected in few samples (mushroom, spinach, and lettuce; Fig. 2a). For an interactive example of the results landing page see https://gnps.ucsd.edu/ProteoSAFe/status.jsp?task=16d14b8efd134fcabe227dd6377db1b9. The “view library hits” will reveal if any of the ~620,000 public GNPS reference spectra with structural annotations match the input data. The mirror plot can be used to assess how well the query matched the reference spectrum. The “View Interactive Tree” link displays a visual representation of the food matches organized according to the GFOP ontology. Spirotetramat is an insecticide that also has low use in the US^14^ and the foods sampled. However, this biocide is mainly used on citrus fruits and grapes and was detected in grapes, oranges, and cherries (Fig. 2a). Enilconazole is a fungicide mainly used on citrus fruits^14^ and was detected in 31% of samples classified as citrus (Fig. 2a). Interestingly, enilconazole is also used as an antifungal in veterinary medicine and was the only of the three biocides searched that was detected in a non-plant (goat cheese) sample. A search for cyanidin (Fig. 2b), a plant pigment with reddish-purple color^15^, returned teas (47% prevalence) and fruits; the highest prevalence was observed in raspberries (86%), blackberries (75%), and strawberries (100%). Vitamin B_5_, a ubiquitous metabolite, was detected in many samples, but had the highest prevalence in animal-based foods and fungi (Fig. 2c). We also searched for an antibiotic known to be used in farmed animals. Tetracycline^16^ (Fig. 2d) was detected in beef (5%) and poultry (22%). Finally, we searched for an unknown compound (Fig. 2e) that was detected in an Alzheimer’s clinical cohort. The unknown had the highest detection rate in rice (27%) and oat (17%) samples, enabling the formulation that it may be associated with dietary habits. Links to the foodMASST jobs described above can be found in the data availability statement.

There are some precautions one must take to prevent the over-interpretation of the results. Additionally, there are limitations with the presented foodMASST approach that are not specific to foodMASST but rather general to MS/MS spectral matching based on untargeted metabolomics. For example, mass spectrometry can be collected in positive and negative ion modes. The reference data is currently limited to positive ion mode and thus molecules only ionizable in negative mode cannot be used, however, this infrastructure can easily accommodate negative ion mode if the community chooses to provide such reference data. Another caveat is that two different molecules, especially structurally related isomers, can have nearly identical MS/MS spectra. Another common feature of mass spectrometry is that molecules may be ionized as different adducts (e.g., H^+^, Na^+^, K^+^, NH_4_^+^). It is common in an untargeted metabolomics experiment to have multiple adducts for each molecule. We encourage searching all adducts that have MS/MS information as it is impossible to get informative MS/MS matching when there are 1, 2, or 3 fragment ions. Such searches provide too little structural information to be reliable^17^ and therefore the use of low information MS/MS spectral entries are discouraged. In general, the more ions and tighter the mass tolerances used for the search the less likely spurious matches are obtained.

The user may also be interested in structural analogs of related molecules in different foods as they are likely to have similar biological activities. Distributions of analogs can be discovered by searching in analog mode and reporting the neighbors of the searched spectrum in a molecular network. Analog searches will also allow improved discovery of MS/MS matches collected on different instruments or with different instrument settings.

The GFOP reference dataset will continue to grow. The community can contribute to the database that foodMASST uses by depositing LC-MS/MS-based metabolomics data, with the food-specific metadata, into GNPS/MassIVE followed by correspondence with the authors who will inspect the contributed data and add it to the existing database. We anticipate foodMASST will provide valuable insight for unknown MS/MS signals relevant to clinical studies and known signals being considered as dietary biomarkers. More broadly, the enhancement of MASST for domain-specific reporting using well-curated reference datasets will undoubtedly prove useful for many research areas.

## Methods

### Reference data

The existing functionality of MASST^3^ was utilized to create a workflow wherein MS/MS matches identified within the GFOP food reference dataset are reported and contextualized. The GFOP reference dataset contains untargeted metabolomics data acquired from over 3500 food and beverage samples encompassing both human and animal dietary components. Each sample is associated with metadata to describe its characteristics, source, and preparation. Samples were also organized according to a custom ontology which, at the highest level, distinguishes between plant- or animal-based foods, algae, fungi, supplements, minerals, and animal feeds. The ontology is stored and managed using WebProtégé (Stanford University, California, USA), and can be viewed at https://webprotege.stanford.edu/#projects/23f59b5a-4c29-41df-b2d8-a2ea7282d912/edit/Classes. Each sample was labeled using the most specific ontology term based on the metadata provided (e.g., one sample might be classified as a red cherry tomato while another might be broadly classified as tomato).

### Reporting of spectral matches

To calculate the proportion of matches at every level of the ontology, each sample inherited all parent terms of its terminal label (e.g., terminal label: red cherry tomato; parent labels: cherry tomato, tomato, berry, fleshy fruit, fruit, plant). For every ontology term, the number of samples with that label and a spectral match to the query was divided by the total number of samples with that label. The GFOP ontology was combined with the foodMASST results and visualized in a tree structure using the D3.js library and code adapted from Rob Schmuecker (https://bl.ocks.org/robschmuecker).

### Reporting summary

Further information on research design is available in the Nature Research Reporting Summary linked to this article.

## Supplementary information

Reporting Summary

## Data Availability

The raw data used in this study are publicly available online at MassIVE (https://massive.ucsd.edu/) under the accession MSV000084900. foodMASST jobs provided as examples can be viewed at: Domoic acid https://gnps.ucsd.edu/ProteoSAFe/status.jsp?task=d77bb006bcbf48f79d789e60c4238899; Spirotetramat https://gnps.ucsd.edu/ProteoSAFe/status.jsp?task=2784cf02f6864321a24390d00f7e3263; Enilconazole https://gnps.ucsd.edu/ProteoSAFe/status.jsp?task=f6759397ec7d4ca7995d239a1a57d2a5; Fenamidone https://gnps.ucsd.edu/ProteoSAFe/status.jsp?task=16d14b8efd134fcabe227dd6377db1b9; Cyanidin https://gnps.ucsd.edu/ProteoSAFe/status.jsp?task=b7eb71d28f52438c87d969c3e9618b9e; Pantothenic acid https://gnps.ucsd.edu/ProteoSAFe/status.jsp?task=6936ab0b89ec4a7aaec79a3a935f9e08; Tetracycline https://gnps.ucsd.edu/ProteoSAFe/status.jsp?task=e5021c2bd8cc4f41ae35da99d5bfe4f2; Unknown (m/z = 457.257) https://gnps.ucsd.edu/ProteoSAFe/status.jsp?task=da0777bdc1eb43aeb60791faf7d36011.

## Code availability

The open-source code for foodMASST is available on GitHub (https://github.com/mwang87/GNPS_MASST and https://github.com/CCMS-UCSD/GNPS_Workflows).
